# Much ado about *p*. What does a *p*-value mean when testing hypotheses with aggregated cross-cultural data in the field of evolution and human behavior?

**DOI:** 10.3389/fpsyg.2013.00734

**Published:** 2013-10-11

**Authors:** Thomas V. Pollet

**Affiliations:** Department of Social and Organizational Psychology, VU University AmsterdamAmsterdam, Netherlands

**Keywords:** pathogen stress, cross-cultural correlation, inferential statistics, *p*-value, cross-cultural research

Several recent papers in the field of Evolution and Human Behavior rely on aggregate data when testing their hypothesis on adaptations in humans. This is perhaps most notably the case for studies on pathogen stress, (e.g., DeBruine et al., 2010; Thornhill and Fincher, 2011; Fincher and Thornhill, 2012). These studies predominantly rely on cross-cultural correlations and present *p*-values in support of their hypotheses. In this opinion article, I demonstrate why *p*-values can be questionable in this context. I do not wish to single out a particular research area, as the misinterpretation of *p* in this context seems relatively widespread. But for the purpose of this opinion article I will largely draw on examples from work relating to pathogen stress, as this research area most prominently appears to rely on aggregated cross-cultural data. I also want to stress that this is not a general critique of *p*-value usage or frequentist statistics (e.g., Johnson, 1999; Anderson et al., 2000; Goodman, 2008; Ziliak and McCloskey, 2008; Wetzels et al., 2011), but rather a critique on the reliance on *p*-values when using macrolevel data in cases where the sample closely matches the entire range of possible observations. This opinion article is also not a critique of reliance on macrolevel data *per se*, or of a research programme in particular, but focuses on one particular aspect: statistical inference from macrolevel data when a sample closely matches the entire population.

## Inferential statistics

As is commonly documented (e.g., Howell, 2010), inferential statistics are used when based on a finite sample set of observations, we want to make statistical inferences on the “population” of observations via comparing these to known statistical distributions (e.g., Spatz, 2007). For example, with a one-sample *t*-test, we can test whether the population mean of adult male heights from Amsterdam differs from a given value (e.g., 170 cm) based on a given set of observations, a sample of Amsterdammers’ heights (see Myers, 2009). The sample of male heights we obtained is compared to a known statistical distribution (*t* or *Z* distribution, in this case). This comparison gives us a *p*-value, which allows to reject the null hypothesis of no statistical difference between the proposed value of 170 cm and the “true” population mean. A *p* < 0.05 thus would allow us to reject the null hypothesis that the population mean is not statistically different from the hypothesized value of 170 cm, at a 5% significance level.

Similarly to a one sample *t*-test, when making statistical inferences on a Pearson correlation coefficient, we aim to reject the null hypothesis of no association between two given variables in the population based on a (randomized) sample drawn from that population. Statistical inference in this case is usually based on the *t* distribution (see Howell, 2010), but alternative modes of inference can be used (e.g., bootstrapping). The null hypothesis we aim to reject here is that the “true” correlation coefficient (ρ) is 0 in the population. When the concomitant *t*-test is significant, we reject this null hypothesis, in favor of the alternative hypothesis that the “true” correlation coefficient is different from 0 [H(a): ρ ≠ 0].

With cross-cultural correlations, researchers attempt to reject the null hypothesis of no association between these variables at a macrolevel level, i.e., *country*, *region*, or *state* level. This is where statistical inference can become problematic: the observations now consist of countries, states, regions, cultures, or other macrolevel units. In general, there is a relatively small, finite number of these units. It is unlikely that there will be more independent observations in the future. This is unlike observations from rolling a dice, for example, where we can continue to roll a dice, and gather ever more observations. As an example, Thornhill and Fincher (2011) present data on 48 US states, while the finite population of observations arguably consists of 50 US states (51 if we are lenient and grant Puerto Rico state status). In this case, the authors have thus sampled 48 out of the possible 50(/51) potential observations. It is unlikely, that there will be more US states for which we want to make statistical inferences. Even if there were, these “new states” would likely not be independent from the existing ones. In addition, it seems unreasonable to assume that the population of observations is anything else than US states, because this is the unit of observation being sampled (and if this is not the unit of observation, then authors should be explicit what the unit is which they are sampling and consequently wish to make statistical inferences about). In short, in this particular case the authors have sampled over 90% of the observable population and in such a case the use of statistical inference can be questioned. Of course, it is still useful to describe the association via a correlation coefficient, but a *p*-value makes little sense, as the sample very closely matches the entirety of the population, which we want to make inferences about. As an analogy, if I sampled 90% of all males in Amsterdam, then it makes little sense to still rely on *p*-values for making statistical inferences on all males from Amsterdam. Of course, it makes good sense to still describe the data via statistics such as the mean, standard deviation, median, etc., but we can question the use of a *p*-value, when a sample very closely matches the population.

In some cases, (for example, Kanazawa, 2006; DeBruine et al., 2011; Eppig et al., 2011), actually *all* US states have been sampled. In such a case, a *p*-value is entirely nonsensical, as the sample *matches* the population of possible observations, i.e., all US states. To return to the analogy of sampling adult males from Amsterdam, in this case we have the full 100% of the population and there simply is no use for statistical inference: the population is fixed and we have sampled *all* of the possible cases. There is no probability. Again, it is still useful to describe the associations found via correlation coefficients, for example, but statistical inference is unwarranted when the entire population is sampled.

The same argument on the value of statistical inference when the sample closely matches the population, holds for other aggregate units such as nation states or countries: the maximum number of countries in the world is finite [193 (United Nations, 2013) to 195 (U.S. Department of State, 2012) or 196 (Taiwan, included), depending on definitions] and if we have sampled close to all of them, then statistical inference makes little sense. Other aggregate units such as geopolitical regions (Hofstede, 2001), cultural units (e.g., Human Relations Area Files, Naroll, 1967; Lagacé, 1974, 1979), cultures (e.g., Standard Cross-Cultural Sample, Murdock and White, 1969), regions are also finite and we should think carefully about what the population of observations consists of in these cases and how a given sample relates to the population. In some cases, such as geopolitical regions [68 in case of Hofstede (2001)], regions [98 for Fincher et al. (2008)] the population of observations should conceivably be less than maximum of 196 states which exist and we can question whether “new” regions will ever exist or whether the number of regions should be treated as the complete population of possible observations.

**Figure 1:**
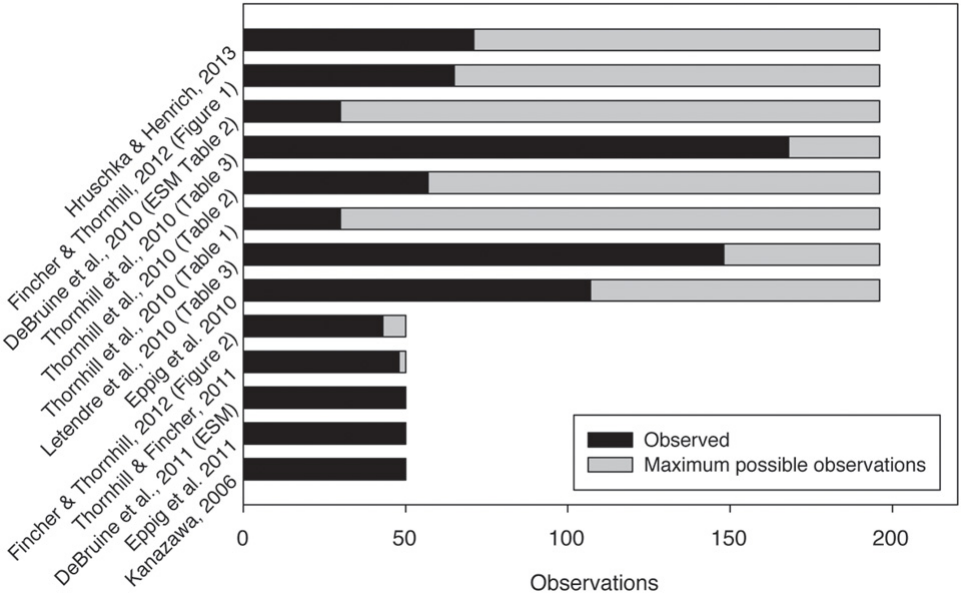
**Some examples of papers with the number of observations and the maximum number of observations. This is a non-exhaustive list of examples to illustrate the issue at hand. [The cases for Hruschka and Henrich (2013) are the lowest *N* used in the paper]**.

Figure 1 shows some examples of the number of observations sampled out of the (reasonable) maximum possible number of observations (data from Kanazawa, 2006; DeBruine et al., 2010, 2011; Eppig et al., 2010, 2011; Letendre et al., 2010; Hruschka and Henrich, 2013). It is by no means a complete set but is merely meant as an illustration of the issue I outlined. As can be seen in some cases, for example DeBruine et al. (2010), a *p*-value could still be useful, when the sample size is relatively small as opposed to the population. However, when there is a large or complete overlap between the number of observations in and the total number of potential observations, we can question the use of a *p*-value.

For ease of comprehension, I have outlined the argument above based on the *p*-value, but the issue I outlined actually already arises with the calculation of the standard error (*SE*) (see Isserlis, 1918; Levy, 2005; Lavrakas, 2008). For finite samples a correction factor should be applied to the *SE*, the finite population correction factor: $\sqrt{\frac{\left(N-n\right)}{\left(N-1\right)}}$, whereby *N* is the population size and *n* is the size of the sample. When this correction factor is not applied, *SE* and therefore *p* is not correctly calculated. In the extreme case when *N* = *n*, this correction factor, and therefore the *SE* in question, will be 0.

A potential reason why some researchers in this area would rely on statistical inference in certain cases, where they should not, could be that they assume that statistical inferences are necessary because their measures (such as IQ for example) have some degree of uncertainty. However, the uncertainty of the measure does not call for statistical inference when all observations are given. Researchers could be mixing up the error associated with measurement for a variable such as IQ at an aggregate level with statistical inference of a relationship *between* aggregate units. Inference of a variable based on a sample (e.g., how well does this sample represent IQ of Wyoming?) is obviously different from inference on the relationship between variables at state level (Is there a statistical relationship between IQ and variable Y at state level?). Regardless of the measurement error of a variable, the “true” statistical relationship is certain when the population is completely sampled: no statistical inference can be made in such a case and therefore *p* is obsolete.

An additional possible reason for incorporating *p*-values when they are unwarranted could be that there are “political” reasons for ritualistically relying on them, perhaps as they provide dichotomous answers to research questions (also see Cohen, 1994; Hoekstra et al., 2006). Editors and reviewers can insist on reporting these *p*-values even when they seem unwarranted and even in the light of frequent calls for alternatives (e.g., Wilkinson, 1999), we continue to heavily rely on them.

In conclusion, in this brief opinion article I questioned the use of *p*-values when the sample consists of aggregate data and the sample of observations closely matches the range of possible observations. I recommend that *p*-values are more critically assessed when applied to macrolevel cross-cultural correlations but acknowledge that there can be many constraints which can lead to their continued usage.
